# Novel Basidiomycetous Alcohol Oxidase from *Cerrena unicolor*—Characterisation, Kinetics, and Proteolytic Modifications

**DOI:** 10.3390/ijms252211890

**Published:** 2024-11-05

**Authors:** Sylwia Stefanek, Rafał Typek, Michał Dybowski, Dorota Wianowska, Magdalena Jaszek, Grzegorz Janusz

**Affiliations:** 1 Department of Biochemistry and Biotechnology, Institute of Biological Sciences, Maria Curie-Skłodowska University, Akademicka 19 St., 20-033 Lublin, Poland; magdalena.jaszek@mail.umcs.pl; 2 Department of Chromatography, Institute of Chemical Sciences, Faculty of Chemistry, Maria Curie-Skłodowska University, Pl. Maria Curie-Skłodowska 3, 20-031 Lublin, Poland; rafal.typek@mail.umcs.pl (R.T.); michal.dybowski@mail.umcs.pl (M.D.); dorota.wianowska@mail.umcs.pl (D.W.)

**Keywords:** *Cerrena unicolor*, alcohol oxidase, proteomic, protease, kinetics

## Abstract

Intracellular alcohol oxidase (AOX) was isolated from the basidiomycetous white rot fungus *Cerrena unicolor* FCL139. The enzyme was semi-purified (13-fold) using two-step chromatography with 30% activity recovery. The identity of the protein was confirmed by LC-MS/MS analysis, and its MW (72 kDa) and pI (6.18) were also determined. The kinetics parameters of the AOX reaction towards various substrates were analysed, which proved that, in addition to methanol (4.36 ± 0.27% of the oxidised substrate), AOX most potently oxidises aromatic alcohols, such as 4-hydroxybenzyl alcohol (14.0 ± 0.8%), benzyl alcohol (4.2 ± 0.3%), anisyl alcohol (7.6 ± 0.4%), and veratryl alcohol (5.0 ± 0.3%). Moreover, the influence of selected commercially available proteases on the biocatalytic properties of AOX from *C. unicolor* was studied. It was proved that the digested enzyme lost its catalytic potential properties except when incubated with pepsin, which significantly boosted its activity up to 123%.

## 1. Introduction

Basidiomycetous fungi have been used for years in biotechnology to obtain new enzymes and other industrially important compounds [1]. Among them, one of the most intensively studied species is *Cerrena unicolor*, a white rot fungus belonging to Basidiomycota that is considered extremely useful for applications in various industries. The most comprehensively studied enzymes obtained from *C. unicolor* are laccase, manganese-dependent peroxidase (MnP), versatile peroxidase (VP), cellobiose dehydrogenase (CDH), and cellulase [2,3,4,5].

Alcohol oxidase [EC.1.1.3.13; AOX] is an enzyme belonging to the glucose-methanol-choline oxidase (GMC) family [6]. It is a homooctameric peroxisomal protein consisting of eight identical subunits ranging from 72 to 79 kDa. Each such subunit is strongly bound by the prosthetic group FAD (flavin adenine dinucleotide) characteristic of this enzyme [7]. AOX is produced mainly by methylotrophic yeasts, such as *Pichia*, *Saccharomyces*, *Candida*, and *Hansenula* (*Ogataea*), and some basidiomycetous fungi [8]. However, AOX from wood-degrading species is hardly described, and its role in wood degradation remains to be elucidated, which is currently the subject of intensive research [6]. A characteristic reaction occurring in yeast, in which AOX participates, is the oxidation of methanol to formaldehyde. However, alcohol oxidase also has the ability to oxidise other alcohols to corresponding aldehydes [9]. In addition to aldehydes, the AOX reaction also produces hydrogen peroxide, which in turn is closely involved in reactions catalysed by peroxidases during wood degradation or is crucial in the Fenton reaction [1]. Interestingly, the AOX from wood-degrading fungi described so far tends to occur not only intracellularly but also outside the mycelium [1,10]. Moreover, the AOX gene is subjected to strong repression by carbon catabolite repression involving a hexose transporter, glucokinase, and hexokinase [11,12,13].

Currently, four groups of AOX are known, namely SCAO-short-chain alcohol oxidase, LCAO-long-chain alcohol oxidase, AAO-aromatic alcohol oxidase, and SAO-secondary alcohol oxidase. Due to their specific properties, all these types of alcohol oxidases can be used in many industries [14]. Due to the oxidation of aliphatic and aromatic alcohols, they can be successfully used in the production of cosmetics, perfumes, and medicines. Nowadays, a domain in the pharmaceutical, medical, and food industries is the quantitative detection and determination of alcohols with high sensitivity and selectivity; hence, AOX can be used in the monitoring of alcohols in food products, analytical chemistry, or forensics [15,16]. AOX is a potentially interesting enzyme as it needs oxygen and oxidises alcohols to aldehydes without reverse reaction [17].

Using AOX to construct specific biosensors seems to be an ideal way to detect medically important compounds such as cholesterol. Another aspect is the production of aldehydes by alcohol oxidase, which has potential applications in perfumery and broadly understood cosmetics [18].

As mentioned, AOX from wood-degrading fungi has been poorly characterised. However, it is highly probable that it participates in the degradation of lignin and may have various catalytic properties. Therefore, the aim of this study was to isolate and characterise the new alcohol oxidase from the biotechnologically important fungus *C. unicolor*. The conducted research consisted of the purification of AOX, identification and analysis of its kinetics properties, and possible proteolytic modification of the enzyme molecule structure.

## 2. Results

### 2.1. Production and Preparation of Alcohol Oxidase

Submerged and aerated culture in the Lindeberg and Holm medium with pH 5.5 was optimal for the growth of *C. unicolor* cultures. Along with the optimisation of the culture time, shaking parameters were also checked. The optimal value of the shaker rotation was 120 rpm. During the studies on time optimisation, the highest value of AOX activity, i.e., 24,900 U/mL, was determined on the 14th day of culture.

### 2.2. Semi-Purification and Identification of Alcohol Oxidase in the Secretome of C. unicolor FCL139 by LC-MS/MS

Alcohol oxidase from *C. unicolor* was semi-purified by means of chromatography (Table 1). During the three steps of purification, which comprised salting-out, HIC, and SEC, the enzyme was purified 13-fold from the crude extract of *C. unicolor* mycelium with a total yield of 30%. Storage had a negative effect on the enzymatic activity of alcohol oxidase. After 7 days of storage at −20 °C, 50% of AOX activity was retained. Similar values were observed during storage at −80 °C and +4 °C.

Extracellular proteins were analysed by LC-MS/MS from a lyophilised preparation of *C. unicolor* AOX. Based on the presence of unique peptides, an alcohol oxidase was identified with a sequence similarity of 76% coverage to the protein with ID number 338999 in the *C. unicolor* v1.1 (Cerun2) genome (DOE JGI, http://jgi.doe.gov, assessed on 17 May 2023). Moreover, the molecular weight was determined in silico to be 72 kDa, and the isoelectric point (pI) was 6.18.

### 2.3. Enzyme Functional Parameters

**Figure 1:**
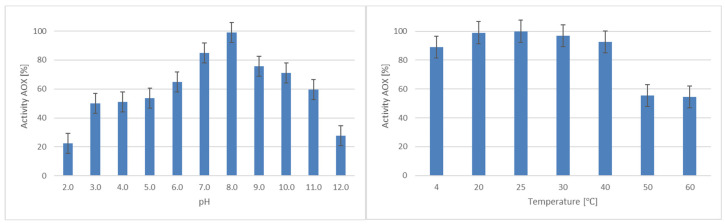
Estimation of the pH (**left**) and temperature (**right**) optima for the reaction of *C. unicolor* alcohol oxidase towards methanol.

The optimal pH and temperature range for the reaction of alcohol oxidase towards methanol was established (Table 2). The appropriate pH range for AOX is 7.0–8.0, and the temperature is 20–30 °C (Figure 1).

The activity of alcohol oxidase towards selected substrates was analysed with a comparison thereof to methanol assumed as 100% (Table 2). Significant activity of the AOX enzyme was observed in the reaction with 4-hydroxybenzyl alcohol, veratryl alcohol, benzyl alcohol, 2-methyl-1-propanol, and L-arabinitol. The values of kinetic constants K_m_ [mM] and V_max_ [µM min^−1^] were analysed in the case of these compounds. The results indicate that AOX from *C. unicolor* had the highest affinity towards methanol, with K_m_ of 12.99 mM when using biochemical assay. Benzyl alcohol was the least preferred substrate, albeit with high V_max_.

Alcohol oxidase activity was measured against selected inhibitors typical for oxidoreductases (Table 3). Significant inhibition properties were also demonstrated by EDTA (0% activity at a concentration of 100 mM). A slight decrease in the AOX activity was observed upon the addition of NaBr at a concentration of 100 mM, as 59 % of the alcohol oxidase activity was retained, and at 50 mM imidazole (24 %); however, the higher concentrations of this compound did not block the enzyme. This was similar in the case of sodium chloride (NaCl), which inhibited the action of AOX at a concentration of 5 mM and retained 87 % of its activity and at a concentration of 100 mM NaCl (only 8%).

### 2.4. Oxidation of Selected Alcohols by AOX Isolated from C. unicolor

According to the results regarding the efficiency of enzymatic oxidation of alcohols, the highest efficiency was achieved for aromatic alcohols. This is not surprising due to the fact that the phenyl group in the *alpha* position relative to -OH activates the oxidation process. Additionally, it should be noted that the aldehyde group formed enters into mesomeric resonance with the aromatic structure, which also favours the oxidation process [19].

A more detailed analysis of the data on the oxidation of aromatic alcohols showed a much greater efficiency of this process in the case of benzyl alcohols substituted in the aromatic ring than in the case of benzyl alcohol. The substituent effect is most visible in the case of 4-hydroxybenzyl alcohol, where the -OH group is in the *para*-position in relation to the emerging aldehyde group.

The effect of the formation of conjugated bonds also occurs in the case of aliphatic alcohols in which the -C=C- bond and mesomeric effect occur in the *beta* position in relation to the -OH group. The comparison of the amounts of unsaturated aliphatic aldehydes formed relative to the analogous saturated forms showed that the amounts of the former were higher (compare propanal and butanal with allyl aldehyde and *cis*-but-2-enal, respectively, in Table 4).

The branching of the aliphatic chain also has a positive effect on the amount of aldehydes produced, as seen in the case of butanal and 2-methyl-propanal (Table 4).

The last conclusion from the analysis of the data contained in the table is the decreasing efficiency of the formation of saturated aldehydes in the homologous series. This behaviour is analogous to the chemical oxidation of alcohols using oxidising reagents resulting from a decrease in the reactivity of alcohols with the extension of the aliphatic chain and is consistent with the literature [19].

### 2.5. Analysis of the Influence of Proteolytic Digestion on the Catalytic Properties of Alcohol Oxidase Isolated from C. unicolor

This analysis consisted of incubation of selected commercially available proteolytic enzymes with alcohol oxidase and then testing its activity (Table 5). The results clearly indicated that all the proteases used had a significant impact on the AOX activity. It may be easily observed that the incubation with pycnoporopepsin resulted in lowering the AOX activity to 61% of the initial level after one hour. In contrast, the addition of pepsin increased the oxidoreductase activity significantly to 126% after 30 min of incubation, and the effect was visible until the end of the experiment (130%). Furthermore, the addition of pepstatin to the pepsin-enzyme mixture weakened this effect, regardless of whether it was subjected to 30 min of incubation. Trypsin and bromomelanin had a lower effect on the AOX activity.

## 3. Discussion

Alcohol oxidase has been previously characterised mainly in methylotrophic yeasts, such *as Pichia pastoris* [20], *Ogataea polymorpha* [21], and *Candida methanosorbosa* [22]. However, there are reports of AOX characteristics of basidiomycete fungi, e.g., *Gleophyllum trabeum* [23] and *Phanerochaete chrysosporium* [24]. It was suggested in the cited papers that yeast developed the ability to metabolise methanol, which is present in their natural niches. However, it seems that wood-degrading fungi may encounter not only this primary alcohol as a result of lignin degradation [25] but also other alcohols, including aromatic ones. Therefore, it was speculated that alcohol oxidase may play an important role in metabolising by-products by wood-degrading fungi. In this study, a novel basidiomycetous alcohol oxidase of intracellular origin from *C. unicolor* was partially purified and characterised. Recently, genes coding for AOX have been proven to be regulated when this fungus was grown on sawdust or in different lighting conditions [26,27]. Therefore, it was assumed that alcohol oxidase may play an important role in *C. unicolor* metabolism, and it might be interesting to analyse its characteristics.

When analysing the *C. unicolor* genome, four genes belonging to the AA3_3 class (alcohol oxidase) of CAZymes were found. As the first step, the identity of alcohol oxidase was confirmed by LC-MS/MS analysis, and it was proved that this protein is a product of the expression of one gene. The molecular weight of AOX is typical for the basidiomycetous intracellular form occurring as an octamer. Similar results were obtained for *Gleophyllum trabeum* or *Phlebiopsis gigantea* [23,28]. Interestingly, in previous studies, four genes were up- and down-regulated when *C. unicolor* was grown on sawdust, as proved by transcriptomic analysis [26]. The pH and temperature optima of semi-purified alcohol oxidase from *C. unicolor* are in the range typical for basidiomycetous or yeast AOX [28,29].

Recent studies have proved that basidiomycetous alcohol oxidase may catalyse the oxidation of not only aliphatic but also aromatic alcohols [30]. In the case of typical alcohol oxidase, the former alcohols are oxidised with decreased efficiency at the growing number of carbon atoms, and aromatic alcohols are more poorly oxidised [23], in contrast to aryl-alcohol oxidase [31].

The *C. unicolor* alcohol oxidase catalysed the oxidation of primary alcohol with decreasing efficiency along the growing chain length. However, it was able to oxidase derivatives of benzyl alcohol with a higher yield than that of methanol, ethanol, and other primary alcohols. The results obtained in the study were proved using two different methods: the biochemical assay and the HPLC analysis.

The ability to oxidase derivatives of benzyl alcohol is interesting, as they are mainly substrates for aryl-alcohol oxidase and even veratryl alcohol is used as a common substrate for the AAO assay [32]. The *C. unicolor* alcohol oxidase showed the highest affinity towards methanol (K_m_ = 12 mM), which is a similar result to that obtained for *Phanerodontia chrysosporium* [33]. The lowest K_m_ (below 1 mM) was determined for alcohol oxidases from yeast [28,34].

The highest V_max_ was found for benzyl and 4-hydroxybenzyl alcohol. This finding is intriguing and may find potential application in the design of specific biosensors used to recognise and distinguish simple alcohols in medicine, pharmacy, or the food industry. In contrast to yeast, it may be an evolutionary advantage, as wood-decomposing fungi encounter aromatic alcohols (besides aliphatic) when degrading lignin. It may also be the reason for the different localisation of alcohol oxidase in cells, even observed as an extracellular protein in basidiomycetous fungi. However, the extracellular form of alcohol oxidase is a product of the expression of a different gene and remains inactive.

The available literature data indicate that the activity of laccase from *C. unicolor* is probably also regulated by commercial proteases [35] and endogenous aspartic protease [36]. Moreover, a peroxisomal protease with trypsin-like activity was proved to be engaged in the maturation of alcohol oxidase in *Candida boidinii* [37]. Monomers of alcohol oxidase (AO), an abundant protein in *C. boidinii* peroxisomes, are sensitive to degradation by this protease, and sensitivity is lost over time in vivo as AOX folds and matures into octamers. Bearing these results in mind, a similar approach was used to analyse the influence of proteases on the *C. unicolor* alcohol oxidase activity. As in the case of laccase studied previously, pepsin increased the alcohol oxidase activity when they were incubated together. In this case, it is worth emphasising that it is probably the result of pepsin catalytic activity towards the alcohol oxidase molecule. This is supported by the fact that the addition of a selective inhibitor of this enzyme (pepstatin) eliminated the boosting effect. In contrast, the other proteases decreased its activity. The observed influence of pepsin on alcohol oxidase activity may be used to obtain higher AOX activities.

## 4. Materials and Methods

### 4.1. Materials

HPLC-grade acetonitrile (ACN), phosphoric (V) acid, and ammonium bicarbonate were supplied by the Polish Chemical Plant Avantor (Gliwice, Poland). 2,4-Dinitrophenylhydrazine (DNPH), Aldehyde/Ketone-DNPH Stock Standard-13, phenylmethylsulfonyl fluoride (PMSF), acetylacetone (3,5-diacetyl-1,4-dihydrolutidine), Tris[2-carboxyethyl]phosphine-HCl (TCEP), and methyl methanethiosulfonate (MMTS) were purchased from Sigma Aldrich (Poznan, Poland). Deionised water was purified on a Milli-Q system from Millipore (Millipore, Bedford, MA, USA).

### 4.2. Fungal Strain and Culture Conditions

The white rot fungus *Cerrena unicolor* FCL139 was obtained from the culture collection of the Regensburg University (Regensburg, Germany) and maintained on 2% (*w*/*v*) malt agar slants. The mycelium was transferred onto plates with malt extract medium and cultured for 7 days in room conditions (temperature 25 °C and daylight).

The Lindeberg and Holm [38] liquid medium and a 1 cm^2^ piece of mycelium were added to a 250 mL Erlenmeyer flask. The inoculum culture was maintained for 8 days at 25 °C. After this time, the mycelium was homogenised (15,500 rpm) using a dispenser (IKA, Warsaw, Poland) in sterile conditions. Then, 10 mL of the homogenised mycelium was added to a 100 mL Erlenmeyer flask with the Lindeberg and Holm medium, placed on a shaker Minitron (Infors HT, Basel, Switzerland) at 120 rpm at 28 °C, and cultured for 16 days. To analyse the dynamics of the AOX synthesis by *C. unicolor* starting from the sixth culture day, mycelial samples were taken, and the activity of alcohol oxidase was assayed. After optimisation of the *C. unicolor* cultivation time, 9 glass flasks with a capacity of 100 mL were prepared, to which 30 mL of Lindeberg and Holm medium and 10 mL of *C. unicolor* inoculum were added. The shake culture was carried out for 14 days, after which time samples were collected and homogenised, and AOX activity was measured. The shaking speed of the culture was optimised for values of 100, 120, and 140 rpm using an Infors HT Minitron shaker.

### 4.3. Enzyme Assay

The enzyme was extracted from the mycelium as in Daniel et al. [23] with one modification, i.e., 2 mg/mL of PMSF (phenylmethylsulfonyl fluoride) was added to the extraction buffer. The acetylacetone method was used to measure alcohol oxidase activity by forming 3,5-diacetyl-1,4-dihydrolutidine as the final product [39]. The enzyme activity was expressed as U/l. The protein concentration was estimated using the Bradford method [40]. For pH optimum analysis, Britton–Robinson buffer was used in the range of 2.0 to 12.0, and temperature optimum was analysed between 4 and 60 °C.

### 4.4. Enzyme Purification

Mycelium from 10-day culture (5 g) was homogenised in phosphate–potassium buffer containing PMSF using a disperser T18 (IKA, Warsaw, Poland) and then centrifuged for 30 min at 16,000× *g*. The resulting supernatant was desalted with ammonium sulphate in the range 40–60% of the final concentration. The obtained precipitated enzyme was pelleted by centrifugation as described above and diluted in 0.1 M sodium phosphate buffer pH 7.5. After further centrifugation, the supernatant was used for hydrophobic interaction chromatography (HIC). For this purpose, the preparation was applied to a 1 mL Sepharose Phenyl FF (LS) column (GE Healthcare, Chicago, IL, USA), and chromatographic separation was performed using sodium phosphate buffers pH 7.5 with the addition of 1.5 M ammonium sulphate. In the next step, the salts were removed from the obtained enzyme using a GoBio Mini Dsalt 5 mL column in the presence of distilled water. An AKTA Prime Plus (GE Healthcare, Chicago, IL, USA) chromatography system was used for both chromatographic separations.

### 4.5. LC-MS/MS Analysis

The purified enzyme samples were freeze-dried in a FreeZone 12 Freeze Dryer (Labconco, Kansas City, MO, USA). An individual sample (20 µg of protein) was suspended in 100 µL of 100 mM ammonium bicarbonate and reduced with 5 mM Tris[2-carboxyethyl]phosphine-HCl (TCEP) for 1 h at 60 °C, blocked with 10 mM methyl methanethiosulfonate (MMTS) for 10 min, and digested overnight with 10 ng/µL mass spectrometry-grade trypsin. The resulting peptide mixture was analysed using UPLC (NanoACQUITY, Waters, Milford, MA, USA) coupled with an Orbitrap Velos mass spectrometer. The resulting output list of precursor and product ions was compared with the protein database of *C. unicolor* (12,978 residues: 5371,935 residues) using the local MASCOT server (version 2.4.1). The *C. unicolor* v1.1 (Cerun2) genome assembly was downloaded from the United States Department of Energy, Joint Genome Institute (DOE JGI, http://jgi.doe.gov, accessed on 16 January 2016) and used as a reference for peptide mapping. The MS/MS analysis was performed at the Environmental Mass Spectrometry Laboratory of Biochemistry and Biophysics of the Polish Academy of Sciences in Warsaw. The protein score cut-off was set at 150.

### 4.6. Enzyme Kinetics

The activity of the purified alcohol oxidase towards selected substrates was measured, and its catalytic potential was examined. Methanol was assumed to be 100% the simplest alcohol in the aliphatic series. The K_m_ and V_max_ values for the purified enzyme were determined using various concentrations of substrates (from 1 mM to 100 mM) by non-linear least squares, fitting the observed data to the Michaelis–Menten equation. The OriginPro 8 software version 95E (OriginLab Corporation, Northampton, MA, USA) was used for data analysis. The effects of various potential inhibitors of AOX activity were investigated by adding these compounds to the final concentration from 1 mM to 100 mM to the samples of the enzyme and then assayed towards methanol as a substrate. Control tests were performed in parallel in the absence of the inhibitor. All measurements were performed in triplicates.

### 4.7. HPLC Analysis of Alcohol Oxidase Catalytic Efficiency

After 1 h enzymatic oxidation of alcohols in a buffering solution at room temperature, the samples were mixed with a derivatisation reagent in a ratio of 1:10 and incubated at room temperature for 1 h. The derivatisation reagent applied in the research was obtained by introducing 100 mg of DNPH into 70 mL of ACN. After the crystalline DNPH was completely dissolved in ACN, 30 mL of a 15% aqueous phosphoric acid (V) solution was added and mixed thoroughly.

In order to estimate the efficiency of the enzymatic transformation of alcohols into aldehydes, derivatised post-process samples were subjected to HPLC analysis. The analytical system used in the research, Nexera-i LC-2040 3D (Shimadzu, Kyoto, Japan), was equipped with a PDA detector operating at 360 nm. A 250 mm × 4.6 mm i.d., 3 μm Synergi Polar-RP column (Phenomenex, Torrance, CA, USA) was used. Chromatographic separation was performed using gradient elution. Water was used as mobile phase A, and acetonitrile served as mobile phase B. The gradient programme started at 35% B, increasing to 95% for 60 min, and finally, isocratic elution followed (95% B) for 10 min. The total run time was 70 min at a mobile phase flow rate of 1 mL/min.

Due to the lack of their standards, the amounts of the DNPH derivatives of 4-hydroxybenzyl, anisyl, and veratryl aldehyde were determined based on the calibration for benzylaldehyde. In turn, the concentration of isobutanal was determined using the calibration for butanal. All results are presented as the mean value of three independent measurements (n = 3) ± SD.

### 4.8. Digestion of AOX by Proteases

A solution of lyophilised AOX (activity 27.1 U/mg of protein) in ultra-pure water was incubated in a ratio 1:1 with a water solution of commercially available pepsin (4.2 U/mg), trypsin (10.3 U/mg), bromelain (7.0 U/mg), and pycnoporopepsin (17.2 U/mg) at 37 °C. Pycnoropepsin was isolated from *Pycnoporus saguineus*, according to Ichishima et al. [41]. Trypsin was supplied from MP Biomedicals (Irvine, CA, USA), and the other enzymes were supplied by Merck Group Sigma Aldrich (Saint Louis, USA). In the case of pepsin, a 30 min preincubation with the inhibitor pepstatin (10 qM) was also performed as a control. The alcohol oxidase activity was assayed periodically at the 0 point (immediately after mixing) and after 30, 60, and 120 min of incubation.

## 5. Conclusions

Alcohol oxidase from *C. unicolor* is a protein with promising catalytic properties and, therefore, can potentially be used in biotechnological applications, e.g., in the synthesis of aromatic aldehydes. The identification of a novel strain capable of AOX synthesis encourages further research focused on scaling-up protein synthesis and optimisation of alcohol oxidation processes. However, further research on enzyme storage and/or isolation is needed.

## Figures and Tables

**Table 1:** Purification of AOX from *C. Unicolor*.

Purification Step	Total Activity [U]	Total Protein [MG]	Specific Activity [U/MG]	Recovery [%]	Purification [FOLD]
Crude extract	20,400	1150	17.7	-	1
Ammonium sulphate purification	21,600	648	33.3	106	1.9
Hydrophobic interaction chromatography [HIC]	19,400	37	523	95	29.6
Size exclusion chromatography [SEC]	6040	26	229	29.6	12.9

**Table 2:** Substrate specificity of alcohol oxidase obtained from *C. unicolor*. Methanol was considered a typical substrate for alcohol oxidase and accounted for 100% of the reaction.

Substrate	Activity AOX [%]	K_m_ [mM]	V_max_ [µM·min^−1^]
Methanol	100	13 ± 1	7500 ± 100
4-hydroxybenzyl alcohol	98.0 ± 0.1	154 ± 12	19,800 ± 200
Veratryl alcohol	50 ± 1	31 ± 4	1680 ± 100
Benzyl alcohol	38.0 ± 0.1	1270 ± 12	85,300 ± 800
2-methyl-1-propanol	34.0 ± 0.6	1040 ± 10	34,500 ± 300
L-arabinitol	12.0 ± 0.3	37 ± 3	1000 ± 100
Allyl alcohol	7.0 ± 0.1		
Cis-2-butanol	5.0 ± 0.8		
Amyl alcohol	4.0 ± 0.1		
1-propanol	2.0 ± 0.2		
Ethanolamine	2.0 ± 0.1		
2-propanol	1.0 ± 0.2		
Isoamyl alcohol	1.00 ± 0.06		
1-butanol	1.0 ± 0.2		
Ethanol	0.50 ± 0.05		
2-butanol	0		
Glycerol	0		
Tetra-butanol	0		
3-methyl-1-butanol	0		

**Table 3:** Effect of potential inhibitors on the activity of alcohol oxidase (AOX). Enzyme activity was expressed in %.

Inhibitor Type	Inhibitor Concentration [MM]					
	0.1	1	5	10	50	100
NaCl	100 ± 5	100 ± 2	87 ± 6	78 ± 2	65.2 ± 0.1	8.4 ± 0.1
NaBr	100 ± 6	100 ± 1	100 ± 5	100 ± 7	62 ± 2	59 ± 1
Imidazole	100.0 ± 0.1	100 ± 3	100 ± 2	100 ± 6	24 ± 2	0
EDTA	90.2 ± 0.8	89.0 ± 0.4	79.1 ± 0.1	65.8 ± 0.6	34 ± 1	0

**Table 4:** Substrate specificity of alcohol oxidase isolated from *C. unicolor*.

No.	Oxidised Substrate	% Conversion to Aldehyde
1	Methanol	4.4 ± 0.3
2	Ethanol	2.8 ± 0.2
3	1-Propanol	1.7 ± 0.1
4	1-Butanol	0.70 ± 0.04
5	1-Pentanole	0.13 ± 0.01
6	2-Methyl-propan-1-ol	1.6 ± 0.1
7	Allyl alcohol	2.2 ± 0.1
8	*cis*-2-butenol	1.5 ± 0.1
9	Benzyl alcohol	4.2 ± 0.3
10	4-Hydroxybenzyl alcohol	14.0 ± 0.8
11	Anisyl alcohol	7.6 ± 0.4
12	Veratryl alcohol	5.0 ± 0.3

**Table 5:** Influence of selected proteolytic enzymes on the AOX activity of *C. unicolor*.

Alcohol Oxidase Activity [%] in Time				
	0 min	30 min	60 min	120 min
Control (AOX + H_2_O 1:1)	100 ± 1	100 ± 2	100 ± 1	100 ± 1
Pepsin + AOX 1:1	83 ± 3	126 ± 2	123 ± 1	123 ± 1
Pepsin + AOX + pepstatin (10 qL) with incubation 30 min 1:1	75.5 ± 0.5	99 ± 2	100 ± 1	95 ± 2
Pepsin +AOX + pepstatin (10 qL) without incubation 1:1	67 ± 2	90 ± 1	86.0 ± 0.2	88 ± 1
Trypsin + AOX 1:1	80.3 ± 0.7	84 ± 1	88 ± 1	92 ± 2
Bromomelanin + AOX 1:1	87 ± 2	104 ± 1	99 ± 1	100 ± 1
Pycnoporopepsin + AOX 1:1	63 ± 1	64 ± 1	61.4 ± 0.3	63 ± 1

## Data Availability

Not applicable.

## References

[1] Roth M.G., Westrick N.M., Baldwin T.T. (2023). Fungal biotechnology: From yesterday to tomorrow. Front. Fungal Biol..

[2] Sulej J., Janusz G., Osińska-Jaroszuk M., Rachubik P., Mazur A., Komaniecka I., Choma A., Rogalski J. (2015). Characterization of Cellobiose Dehydrogenase from a Biotechnologically Important Cerrena unicolor Strain. Appl. Biochem. Biotechnol..

[3] Perna V., Agger J.W. (2023). Transesterification with CE15 glucuronoyl esterase from Cerrena unicolor reveals substrate preferences. Biotechnol. Lett..

[4] Yao Y., Zhou G., Lin Y., Xu X., Yang J. (2020). A highly thermotolerant laccase produced by Cerrena unicolor strain CGMCC 5.1011 for complete and stable malachite green decolorization. AMB Express.

[5] Belova O.V., Lisov A.V., Vinokurova N.G., Kostenevich A.A., Sapunova L.I., Lobanok A.G., Leontievsky A.A. (2014). Xylanase and cellulase of fungus Cerrena unicolor VKM F-3196: Production, properties, and applications for the saccharification of plant material. Appl. Biochem. Microbiol..

[6] Pawlik A., Stefanek S., Janusz G. (2022). Properties, Physiological Functions and Involvement of Basidiomycetous Alcohol Oxidase in Wood Degradation. Int. J. Mol. Sci..

[7] Mangkorn N., Kanokratana P., Roongsawang N., Laosiripojana N., Champreda V. (2018). Purification, characterization, and stabilization of alcohol oxidase from Ogataea thermomethanolica. Protein Expr. Purif..

[8] Maleknia S., Ahmadi H., Norouzian D. (2011). Immobilization of Pichia pastoris cells containing alcohol oxidase activity. Iran. J. Microbiol..

[9] Koch C., Neumann P., Valerius O., Feussner I., Ficner R. (2016). Crystal Structure of Alcohol Oxidase from Pichia pastoris. PLoS ONE.

[10] Sibirny A.A., Titorenko V.I., Efremov B.D., Tolstorukov I.I. (1987). Multiplicity of mechanisms of carbon catabolite repression involved in the synthesis of alcohol oxidase in the methylotrophic yeast *Pichia pinus*. Yeast.

[11] Roggenkamp R., Janowicz Z., Stanikowski B., Hollenberg C.P. (1984). Biosynthesis and regulation of the peroxisomal methanol oxidase from the methylotrophic yeast Hansenula polymorpha. Mol. Genet. Genom..

[12] Stasyk O.V., Stasyk O.G., Komduur J., Veenhuis M., Cregg J.M., Sibirny A.A. (2004). A Hexose Transporter Homologue Controls Glucose Repression in the Methylotrophic Yeast Hansenula polymorpha. J. Biol. Chem..

[13] Kramarenko T., Karp H., Järviste A., Alamäe T. (2000). Sugar repression in the methylotrophic yeastHansenula polymorpha studied by using hexokinase-negative, glucokinase-negative and double kinase-negative mutants. Folia Microbiol..

[14] Goswami P., Chinnadayyala S.S.R., Chakraborty M., Kumar A.K., Kakoti A. (2013). An overview on alcohol oxidases and their potential applications. Appl. Microbiol. Biotechnol..

[15] Stasyuk N., Demkiv O., Gayda G., Zakalska O., Nogala W., Gonchar M. (2022). Amperometric biosensors based on alcohol oxidase and peroxidase–like nanozymes for ethanol determination. Microchim. Acta.

[16] Bucur B., Radu G.L., Toader C.N. (2007). Analysis of methanol–ethanol mixtures from falsified beverages using a dual biosensors amperometric system based on alcohol dehydrogenase and alcohol oxidase. Eur. Food Res. Technol..

[17] Wijayanti S.D., Tsvik L., Haltrich D. (2023). Recent Advances in Electrochemical Enzyme-Based Biosensors for Food and Beverage Analysis. Foods.

[18] Ribeaucourt D., Bissaro B., Lambert F., Lafond M., Berrin J.-G. (2022). Biocatalytic oxidation of fatty alcohols into aldehydes for the flavors and fragrances industry. Biotechnol. Adv..

[19] McMurry J. (2012). Organic Chemistry.

[20] Couderc R., Baratti J. (1980). Oxidation of Methanol by the Yeast, *Pichia pastoris*. Purification and Properties of the Alcohol Oxidase. Agric. Biol. Chem..

[21] Shleev S.V., Shumakovich G.P., Nikitina O.V., Morozova O.V., Pavlishko H.M., Gayda G.Z., Gonchar M.V. (2006). Purification and characterization of alcohol oxidase from a genetically constructed over-producing strain of the methylotrophic yeast Hansenula polymorpha. Biochemistry.

[22] Suye S.-I. (1997). Purification and Properties of Alcohol Oxidase from Candida methanosorbosa M. Curr. Microbiol..

[23] Daniel G., Volc J., Filonova L., Plíhal O., Kubátová E., Halada P. (2007). Characteristics of *Gloeophyllum trabeum* Alcohol Oxidase, an Extracellular Source of H_2_O_2_ in Brown Rot Decay of Wood. Appl. Environ. Microbiol..

[24] Linke D., Lehnert N., Nimtz M., Berger R.G. (2014). An alcohol oxidase of Phanerochaete chrysosporium with a distinct glycerol oxidase activity. Enzym. Microb. Technol..

[25] Ander P., Eriksson K.-E. (1985). Methanol formation during lignin degradation by Phanerochaete chrysosporium. Appl. Microbiol. Biotechnol..

[26] Janusz G., Mazur A., Wielbo J., Koper P., Żebracki K., Pawlik A., Ciołek B., Paszczyński A., Kubik-Komar A. (2018). Comparative transcriptomic analysis of Cerrena unicolor revealed differential expression of genes engaged in degradation of various kinds of wood. Microbiol. Res..

[27] Pawlik A., Mazur A., Wielbo J., Koper P., Żebracki K., Kubik-Komar A., Janusz G. (2019). RNA Sequencing Reveals Differential Gene Expression of *Cerrena Unicolor* in Response to Variable Lighting Conditions. Int. J. Mol. Sci..

[28] Danneel H.-J., Reichert A., Giffhorn F. (1994). Production, purification and characterization of an alcohol oxidase of the ligninolytic fungus Peniophora gigantea. J. Biotechnol..

[29] Leonovich O.A., Kurales Iu A., Dutova T.A., Isakova E.P., Deriabina Iu I., Rabinovich Ia M. (2009). The regulation of peroxisomal matrix enzymes (alcohol oxidase and catalase) formation by the product of the gene Mth1 in methylotrophic yeast Pichia methanolica. Prikl. Biokhim. Mikrobiol..

[30] Bringer S., Sprey B., Sahm H. (1979). Purification and Properties of Alcohol Oxidase from Poria contigua. Eur. J. Biochem..

[31] Guillén F., Martínez A.T., Martínez M.J. (1992). Substrate specificity and properties of the aryl-alcohol oxidase from the ligninolytic fungus *Pleurotus eryngii*. Eur. J. Biochem..

[32] Asada Y., Watanabe A., Ohtsu Y., Kuwahara M. (1995). Purification and Characterization of an Aryl-alcohol Oxidase from the Lignin-degrading Basidiomycete *Phanerochaete chrysosporium*. Biosci. Biotechnol. Biochem..

[33] Nguyen Q.-T., Romero E., Dijkman W.P., de Vasconcellos S.P., Binda C., Mattevi A., Fraaije M.W. (2018). Structure-Based Engineering of *Phanerochaete chrysosporium* Alcohol Oxidase for Enhanced Oxidative Power toward Glycerol. Biochemistry.

[34] Yamada H., Shin K.-C., Kato N., Shimizu S., Tani Y. (1979). Purification and Characterization of Alcohol Oxidase fromCandida25-A. Biosci. Biotechnol. Biochem..

[35] Janusz G., Jaszek M., Matuszewska A., DrĿczkowski P., Osiſska-Jaroszuk M. (2015). Proteolytic modifications of laccase from Cerrena unicolor. J. Mol. Catal. B Enzym..

[36] Pawlik A., Ciołek B., Sulej J., Mazur A., Grela P., Staszczak M., Niścior M., Jaszek M., Matuszewska A., Janusz G. (2021). *Cerrena unicolor* Laccases, Genes Expression and Regulation of Activity. Biomolecules.

[37] Stewart M.Q., van Dijk R., Veenhuis M., Goodman J.M. (2001). Monomeric alcohol oxidase is preferentially digested by a novel protease from Candida boidinii. Biochim. Biophys. Acta (BBA) Mol. Cell Res..

[38] Lindeberg G., Holm G. (1952). Occurrence of Tyrosinase and Laccase in Fruit Bodies and Mycelia of some Hymenomycetes. Physiol. Plant..

[39] Venkatesagowda B., Dekker R.F. (2019). A rapid method to detect and estimate the activity of the enzyme, alcohol oxidase by the use of two chemical complexes-acetylacetone (3,5-diacetyl-1,4-dihydrolutidine) and acetylacetanilide (3,5-di-N-phenylacetyl-1,4-dihydrolutidine). J. Microbiol. Methods.

[40] Bradford M.M. (1976). A rapid and sensitive method for the quantitation of microgram quantities of protein utilizing the principle of protein-dye binding. Anal. Biochem..

[41] Ichishima E., Kumagai H., Tomoda K. (1980). Substrate specificity of carboxyl proteinase fromPycnoporus coccineus, a wood-deteriorating fungus. Curr. Microbiol..

